# Understanding Health, Subjective Aging, and Participation in Social Activities in Later Life: A Regional Finnish Survey

**DOI:** 10.1177/07334648231214940

**Published:** 2023-12-01

**Authors:** Heli Vaartio-Rajalin, Fredrik Snellman, Ylva Gustafsson, Auvo Rauhala, Emilia Viklund

**Affiliations:** 1 Turku University of Applied Sciences/Master School, Turku, Finland; 2 Faculty of Education and Welfare Studies, Department of Health Sciences, 1040 Åbo Akademi University, Vaasa, Finland; 3 Department of Social Work, Faculty of Social Sciences, 8075 Umeå University, Sweden; 4 Faculty of Humanities, Department of Philosophy, 1040 Åbo Akademi University, Turku, Finland; 5 Finnish Centre for Client and Patient Safety, Wellbeing Services County of Ostrobothnia, Vaasa, Finland

**Keywords:** health, perception, population aging, self-concept, participation

## Abstract

To understand health and well-being in later life, it is vital to consider the meaning of subjective aging. This study aimed to explore how perceived health, self-perceptions of aging, and participation in social activities relate to each other among older persons in the Bothnia region and Åland islands in Finland. Data were analyzed using Spearman’s and polychoric correlation and multinomial logistic regression analyses. The perceived good health and the younger physical, psychological, and social dimensions of subjective age were found to be associated with each other and with participation in social activities outside one’s home.

**What this paper adds**

• Younger chronological age, living in relation with someone, and participation into social activities outside home have strong associations with good perceived health
• Perceiving oneself physically younger has a statistically significant association with perceived good health and with participation into social activities outside home
• Older person´s participation into social activities outside home is associated with perceived good health and with younger physical, psychological, and social dimensions of subjective age when compared with chronological age

**Applications of study findings**

• The chronological age and the objective measurements of health status might not be ideal when studying aging and health within gerontological research—different subjective dimensions of age should be considered to a greater extent
• The gap between chronological and subjective age might vary depending on which dimension of subjective age is reflected
• In order to ensure healthy aging and to minimize inequality as well as ageism, it is important to deepen the professional knowledge regarding how the experiences of health and age might influence engaging in activities outside one’s home

## Introduction

Finland is experiencing the most significant population aging, not only in the Nordics but also in a European context, where the country stands out with one of the oldest populations in the region (Nordic Welfare Center, 2022). Several policies have been developed to suggest ways in which long, healthy, and good lives can be achieved (e.g., World Health Organization [WHO], 2017). Although one of the most recent concepts used within aging and health policies, “healthy aging,” (e.g., WHO, 2021), can be seen as a more nuanced picture of what constitutes a good later life than earlier concepts such as successful aging, healthy aging policies have a strong emphasis on biomedical perspectives of functional ability and it can be argued to fail to capture the subjective experiences of the aging process. A person’s own evaluation of their age has been suggested in previous studies to be associated with various health outcomes (e.g., Spuling et al., 2013), and studies report that many older persons seem to feel younger than their chronological age (Wettstein et al., 2023). One potential pathway through which subjective experiences of age might influence self-rated health is by influencing the engagement in everyday activities that can be considered health-promoting (Montepare, 2020). Studies exploring the everyday activities of older persons are warranted in order advance knowledge of subjective aging (Hughes & Touron, 2021). This study aims to deepen the knowledge regarding subjective age, perceived health, and participation in social activities among older persons.

## Background

Subjective aging is an umbrella term covering various and related concepts capturing different age-related experiences, such as age identity, awareness of age-related changes, subjective age, and self-perceptions of aging (Montepare, 2019) and can be defined as a person’s own evaluation of their age and the aging process (Levy et al., 2002). It has been argued to reflect and individual’s cultural and personal perceptions of age and aging (Laslett, 1989). Overall, it has been suggested that concepts focusing on subjective aging better capture the physical, social, and psychological experiences of aging than chronological age (Montepare, 2019). The literature base suggests that a gap between a person’s chronological age and subjective age is not uncommon, and that the discrepancy increase with age (Bergland et al., 2014).

Considering subjective aging seems to be necessary to understand health and well-being in later life, as a large number of studies suggest that self-perceptions of age and aging are related to different aspects of health. For instance, older subjective age has been connected to higher mortality risk (Uotinen et al., 2005; Stephan, et al., 2018) and in a longitudinal study, subjective age was found to predict physical, mental, and perceived health (Spuling et al., 2013). Feeling younger than chronological age has further also been associated with a lower risk of depression, as well as higher levels of mental well-being (Keyes & Westerhof, 2012) and better memory performance (Stephan, et al., 2016). Taken together, previous studies present robust evidence that subjective age influences our multidimensional health.

Additionally, a systematic review by Tully-Wilson et al. (2021) concludes that a more positive outlook on aging is associated with healthier longitudinal outcomes, including better self-rated health and better performance of the activities of daily living. Self-perceptions of aging also seem to influence the health of older couples (Cohn-Swartch et al., 2021), with their own perceptions influencing their own and their partners’ physical and mental health. Positive self-perceptions of aging have been associated with engaging in different types of activities related to exercise (walking, gym, and yoga), but also activities related to appearance (clothes shopping, manicure, and hairdresser/barber) and social activities (visiting friends and family) (Montepare, 2019)., A younger perceived age was furthermore associated with greater participation in social activities among older persons in Japan (Takatori, et al., 2018). In turn, negative perceptions of aging have been suggested to put individuals at risk of social withdrawal, which may increase loneliness and depressive symptoms (Segel-Karpas et al., 2022).

Taken together, current and former policies on aging and health often outline single pathways to health in later life, thus failing to recognize the diversity of the older population (Timonen, 2016) and focusing on functional ability (Nordmyr et al., 2020) rather than other dimensions that are equally important for experiencing health. According to the literature review in this chapter, subjective aging seem to be associated with health, including perceived health in later life, as well as participation in social activities. However, the social dimensions of health have received less attention in studies of subjective aging (Tully-Wilson, et al., 2021). The aim of this study is to explore how perceived health, subjective age, and participation in social activities relate to each other among older persons in the Bothnia region and Åland islands in Finland.

## Methods

The study is reported according to good practice in the conduct and reporting of survey research (Kelley et al., 2003).

### Study Design, Data Material, and Study participants

This study is based on a cross-sectional survey study, conducted within the GERDA project, an interdisciplinary collaboration between Åbo Akademi University, Novia University of Applied Sciences, Seinäjoki University of Applied Sciences and Åland University of Applied Sciences in Finland, and Umeå University in Sweden. The aim of the questionnaire is to gather information on health and living conditions among older persons in the Bothnia region (Finland and Sweden). The latest version of the questionnaire encompassed 92 items covering different themes related to background, housing and living environment, everyday life, and various aspects of health.

Data were collected in regions of Finland with a higher share of Swedish-speaking people. The sampling procedure in 2021 used identical criteria as previous data collections in 2005, 2010, and 2016 with an aim to enable longitudinal follow-up studies of the regional older population. Earlier data collections were funded by the Interreg Botnia Atlantica program (European Regional Development Fund) and thus conditioned by its geographical definition of regions.

The regional EU funding enabled a range of empirical studies, including the one at hand, about the Swedish-speaking minority in Finland, which in small national data samples is rarely represented with an adequate number of individuals for reaching statistical power. Similarly, the sampling procedure also aimed to capture older people in rural areas of the region, who would otherwise not have been reached without adapting a selective sampling technique.

The paper questionnaire was sent out to older adults living in Ostrobothnia (Finland), South Ostrobothnia (Finland), and Åland islands (Finland), that is, in regions where there are both Swedish-speaking and Finnish-speaking populations, in late fall 2021 to every individual born in 1930, 1935, 1940, 1945, 1950, and 1955, except for those living in the city of Vaasa (Ostrobothnia, Finland) and in South Ostrobothnia, where every second or third individual was selected. The participants were selected from the digital and population data services agency in Finland. Only community-dwelling individuals were selected to the sample, meaning that older adults living in effective service houses, hospitals, or nursing homes were excluded. To increase the response rate, the same questionnaire was sent out a second time a few weeks after.

The research ethical principles of the WMA Declaration of Helsinki (2013, https://www.wma.net/policies-post/wma-declaration-of-helsinki-ethical-principles-for-medical-research-involving-human-subjects) were followed. The completed questionnaire was interpreted as participants’ written informed consent. In Finland, ethical approval is not mandatory for anonymous population-based postal surveys (Medical Research Act 488/1999; http://www.finlex.fi/en/laki/kaannokset/1999/en19990488). The plan for data collection and storage was approved by the Swedish Ethical Review Authority the Swedish Ethical Review Authority 2021-04965.

### Measurements and Variables

#### Perceived Health

A variable was created from a one-item question (“In general, how would you describe your health?” with the response alternatives “Excellent,” “Very good,” “Good,” “Fair,” and “Bad”) was used. In this study, the perceived health was treated as an ordinal scale variable with levels from 1 to 5, corresponding to the options from “Bad” to “Excellent.” In regression analyses, a version with merely three levels was used, corresponding from 1 to 3 to the options “Worse than good,” “Good,” and “Better than good.”

#### Subjective age

A variable measuring subjective age was created from a one item-question. Participants were encouraged to evaluate whether they viewed themselves as “younger than I am” [i], “the age I am” [ii], or “older than I am” [iii], related to three different statements, respectively: “I physically feel like [i–iii]” (physical dimension), “I believe other people see me as [i–iii]” (social dimension), and “Inside myself I feel [i–iii]” (psychological dimension). These three statements were considered to capture different dimensions of subjective age, within this study referred to as physical dimension, psychological dimension, and social dimension of subjective age. In correlation and regression analyses, a modification of this ordinal scale variable was used, where the three levels from 1 to 3, corresponded to the options “older than I am,” “the age I am,” and “younger than I am.”

### Participation in Social Activities

Participation in social activities outside the home area (or with someone) was measured by a composite variable that was the number of all such activities of those 10 that each participant had reported having been engaged in. In the same way, participation in activities at home (or alone) was measured by a composite variable of the number of such 12 activities.

### Sociodemographic Variables

Additionally, a set of sociodemographic variables were added to the analysis: age, gender, marital status, mode of living, living region, type of residential area, native language, and level of education (see Table 1). In descriptive statistics, the original classes of these variables were used. For inferential statistics, the variables gender, marital status, type of residential area, and native language were dichotomized to dummy variables presented in Table 2. Age, divided in five years groups 65, 70, 75, 80, 85, and 90 years, was in inferential statistics treated as an interval variable with values from 1 to 6. The level of education was in regression analysis recoded into five classes from 1 to 5 as follows: “Public school” (reference), “Folk high school,” “Lower vocational school,” “Matriculation exam,” and “University exam.” Living region was divided into following three dummy variables, all of which had Southern Ostrobothnia as reference: Swedish Ostrobothnia, Finnish Ostrobothnia, and Åland Islands.

**Table 1.** Background Information of the Participants, *n* = 6926.

Background Variable	*N* (%)
Age (missing 15)	
65	1745 (25)
70	1819 (26)
75	1690 (24.5)
80	836 (12)
85	562 (8)
90	259 (4)
Gender (missing 32)	
Female	3829 (55.5)
Male	3046 (44)
Don’t want to choose	19 (.3)
Marital status (missing 54)	
Married	4469 (65)
Cohabited	392 (6)
Divorced	442 (6)
Unmarried	300 (4)
Widow	1152 (17)
In a relationship, but lives separately	117 (2)
Mode of living (missing 40)	
Town house	4629 (72)
Terraced house	861 (12.5)
Condominium	1230 (18)
Senior apartment	56 (1)
Service apartment	51 (1)
Other	59 (1)
Living region (missing 0)	
Ostrobothnia	3363 (48.5), response rate 46%
Southern Ostrobothnia	2723 (39), response rate 46%
Åland Islands	831 (12), response rate 62%
Type of residential area (missing 142)	
City	3159 (47)
Suburban area/conurbation	707 (10)
Countryside	2840 (42)
Archipelago	78 (1)
Native language (missing 40)	
Finnish	4004 (58)
Swedish	2850 (41)
Other	30 (.4)
Educational degree (missing 38)	
Public school	1918 (28)
Middle school	514 (7.5)
Folk high school	265 (4)
Lower vocational school	1820 (26)
Higher vocational school	1182 (17)
Matriculation exam	346 (5)
University exam	843 (12)

**Table 2.** Polynomial logistic Regression Models for Perceived Health and the Three Types of Subjective.

Dependent Variable	Perceived Health		Physical Dimension of Subjective Age		Psychological Dimension of Subjective Age		Social Dimension of Subjective Age	
	Worse Than Good vs. Good	Better Than Good vs. Good	Older Than I Am vs. True Age	Younger Than I Am vs. True Age	Older Than I Am vs. True Age	Younger Than I Am vs. True Age	Older Than I Am vs. True Age	Younger Than I Am vs. True Age
Overall model *p*	***		***		***		***	
Nagelkerke R^2^, all independent vars. included	.224		.187		.092		.079	
Nagelkerke R^2^, only vars. with *p* < .05 included	.223		.186		.091		.078	
Independent variables	OR (95% CI)^a^	OR (95% CI)	OR (95% CI)	OR (95% CI)	OR (95% CI)	OR (95% CI)	OR (95% CI)	OR (95% CI)
Gender								
Female vs. male (ref.)	NS	NS	1.36 (1.01–1.84)*	NS	NS	1.26–(1.09–1.45)**	NS	NS
Age^b,c^	1.34 (1.25–1.44)***	.88 (.82–.94)***	.68 (0.60–0.76)***	NS	NS	.89 (0.84–0.94)***	0.79 (0.69–0.91)***	1.22 (1.15–1.29)***
Marital status								
In relation vs. not in relation (ref.)	NS	1.40–(1.15–1.71)**	NS	.80 (0.67–0.96)*	NS	NS	NS	NS
Type of residential area								
Urban vs. sparsely populated area (ref.)	.74 (0.61–0.89)**	NS	NS	NS	NS	NS	NS	1.23 (1.06–1.42)**
Native language								
Swedish vs. Finnish (ref.)	.65 (0.54–0.79)***	.37 (.31–.44)***	NS	1.54 (1.32–1.80)***	NS	1.58 (1.37–1.82)***	NS	NS
Education (ref: public school)								
Folk high school	NS	NS	NS	NS	NS	NS	NS	NS
Lower vocational school	NS	NS	NS	NS	NS	NS	NS	NS
Matriculation exam	NS	1.45 (1.01–2.09)*	NS	NS	NS	NS	NS	NS
University exam	.65 (0.48–0.89)**	1.39 (1.07–1.80)*	NS	NS	NS	NS	NS	NS
Number of activities outside the home area^c^	.84 (0.79–0.88)***	1.10 (1.05–1.16)***	.82 (0.75–0.90)***	1.11 (1.06–1.16)***	.84 (0.74–0.95)**	1.06–(1.02–1.10)**	0.86 (0.77–0.96)**	1.11 (1.07–1.16)***
Number of activities in the home area^c^	NS	NS	.91 (0.84–0.99)*	1.07 (1.02–1.11)**	NS	1.05 (1.01-1.09)*	NS	1.05 (1.01–1.09)*
Physical dimension (ref: same as true age)								
Older than true age	6.67 (4.39–10.12)***	.38 (0.18–0.79)*	—					
Younger than true age	.50 (0.40-0.63)***	3.23 (2.72–3.83)*	—					
Perceived health (ref: “good”)								
Better than good	—		.39 (0.19–0.81)*	3.22 (2.71–3.81)***	NS	1.67 (1.41-1.96)***	0.50 (0.29–0.87)**	2.09 (1.77–2.46)***
Worse than good	—		6.59 (4.34–10.00)***	.49 (0.39–0.62)***	4.37 (2.50–7.65)***	0.67 (0.56-0.80)***	2.48 (1.65–3.74)***	NS

^a^Odds Ratio with its 95% Confidence Intervals.

^b^in units of five years.

^c^OR for every additional number or class.

^d^Scale from 1 (poor) to 5 (excellent).

NS, *p* < .05, *p ≤ .05, **p ≤ .01, ***p ≤ .001.

### Analysis

Statistical analyses were conducted with jamovi (R Core Team, 2021; The Jamovi Project, 2022). In descriptive analyses, categorical variables were presented with frequencies and/or percentages. Missing data were not imputed. They were deleted pairwise in correlations and listwise in regressions. All percentages were valid, that is, without including missing data. Pairwise correlations between variables were analyzed using Spearman’s method, except those between the three dimensions of subjective ages and perceived health, where polychoric correlation was used. Regression models were produced using multinomial logistic regression. The perceived health and three dimensions of subjective age each served as the dependent variable. For perceived health, OLS and ordinal regression were also tested. Odds ratios (ORs) and their 95% confidence intervals (CIs) were estimated for independent variables, and *p*-values were presented with levels ⩽.05, ⩽.01, and ⩽.001. Nagelkerke pseudo R^2^ was used as a coefficient of determination. Multicollinearity was tested and not detected. To avoid multicollinearity between region and native language variables that correlated strongly with each other, only one of these two was included in the model at a time.

### Results

Altogether, 6926 older persons (65 years or older) living in their private homes (97%) answered this survey. The response rate varied between the geographical regions in which they live (Table 1).

The majority of participants were 65–81 years old (76%), female (55.5%), and lived in a relationship (71%) in a town or in a suburban area (57%). Participants identified themselves mainly as Finnish-speaking (58%), Swedish-speaking (42%), or other native language-speaking (.4%). The majority of participants had an educational degree from a vocational school or lower (65%).

#### Perceived Health

Of the participants, 8% perceived their health as excellent, 29% as very good, 30% as good, 30% as rather good, and 3% as rather poor. Missing values were 112 of 6926.

The variables associated with perceived health were analyzed using several multinomial logistic regression models. OLS and ordinal regression methods were also used for testing the associations and the results were fairly similar with all three methods. When all independent variables were first placed in the model, Nagelkerke R^2^ was .224, and when those with nonsignificant *p*-values were removed it still reached .223 (Table 2). Better perceived health was found to have a positive relationship with higher level of education (dummy coded levels: “matriculation” or “university examinations” vs. “public school”), lower age, perceiving one’s physical dimension of subjective age as younger compared to chronological age, the number of activities participated outside the home area, and living in a relation. Conversely, living in a sparsely populated area was associated with poorer health. Finnish as native language showed a positive relationship to both worse and better than good health, because those with Swedish as native language tended to have their answers more concentrated in the middle of the scale (which was also the reference) than those with Finnish as native language. In a simple regression model, with only one independent variable, the physical dimension of subjective age alone and activities outside the home area alone raised Negelkerke R^2^ to .12 and to .073, correspondingly.

When the physical dimension of subjective age was changed to the psychological dimension, Nagelkerke R^2^ decreased to .156 and when it was further changed to the social dimension, it remained at the same level.

#### Physical, Psychological, and Social Dimensions of Subjective Age

The participants perceived their physical dimension of subjective age as old as they were in chronological age (59.6%), younger than their chronological age (34.5%), and older (5.8%). Missing values of physical, psychological, and social dimensions of subjective age were correspondingly 222, 282, and 360 of 6926.

According to a multinomial logistic regression model (Table 2), the following independent variables were associated with a younger physical subjective age as the dependent variable: native Swedish language, living not in a relation (as marital status), participating in numerous activities both outside and in the home area, and perceived own health better than average. Older physical dimension of subjective age as the dependent variable was associated with the same variables, except native language and marital status. In addition, older physical dimension of subjective age had a negative relationship with older chronological age. With these variables, the model Nagelkerke R^2^ was calculated to be .186. In a simple regression model perceived own health and activities outside the home area raised correspondingly Negelkerke R^2^ to .144 and to .051.

The participants’ psychological dimension of subjective age was perceived as old as they were (46%), younger (50.5%), or older (3.5%). In a multinomial logistic model with this dependent variable and with only those independent variables that explained the dependent variable, Nagelkerke R^2^ was only .092. The independent variables associated with feeling their psychological dimension of subjective age as younger were mostly the same as for the physical dimension of subjective age except that women more often perceived themselves physically older but psychologically younger than actual age. In a simple regression model perceived own health and activities outside the home area raised Negelkerke R^2^ correspondingly to .070 and to .030.

Regarding the social dimension of subjective age the participants thought that other persons estimated their age as old as they were (56.4%), as younger (39.4%), or as older (4.2%). In a multinomial logistic model those independent variables that were associated with feeling their social dimension of subjective age as younger were again mostly the same than with physical dimension of subjective age, but with these variables Nagelkerke R^2^ was only .078. In addition, those who lived in urban area, perceived more often their social dimension of subjective age as younger.

##### Participation in Social Activities

Regarding participation in different social activities, there were some differences between activities at home (or alone) and activities outside the home area (or with someone, Table 3). About 30% of participants enjoyed leisure activities, such as listening to music, watching TV, doing household work, or gardening, and about 20% doing physical exercises, making food, doing hobbies such as crafting, or playing games. These are examples of activities at home. In addition, participation in activities outside the home area was also identified, and the most popular activities in that group were outdoor living, discussions, and traveling.

**Table 3.** Participation Into Activities at Home and Activities Outside Home Area.

Activity	Participates (percentages, %)	Don’t´Participate but Wishes to Participate (percentages)
Activities at home area (or alone)		
Enjoying music	38	2
Looking tv, videos	36	0.2
Household work	32	1
Gardening	29	3
Making physical exercises	21	7
Making food	18.5	1
Hobbies like crafting, collecting stamps	18	4
Playing games	17	1
Agriculture, forestry	15	0.1
Fishing, hunting	12	1
Having pets	9.5	4
Watching sport	8	5
Activities outside home area (or with someone)		
Outdoor living	37	4
Discussions	33	1
Traveling	27	9
Belong to associations	18.5	2
Cultural activities	16	8
Sailing, bathing	14	2
Entertainment	14	4
Religious activities	10	3
Facility sport	1	0.5
Boll games	1.5	2

According to the regression model, participation in social activities outside home was associated positively with the dependent variable perceived health and with all three types of subjective age, as described above (see Table 2). In simple regression models, participation in social activities outside home was the second greatest predictor of all four dependent variables and much greater than activities in home area.

Native language and region were strongly correlated, because two of the regions were almost entirely Swedish and two Finnish. When the native language was replaced by three region dummy variables in four regression models, also the ORs remained consistent with the language of the regions and R^2^ rather unchanged.

**Figure 1.**
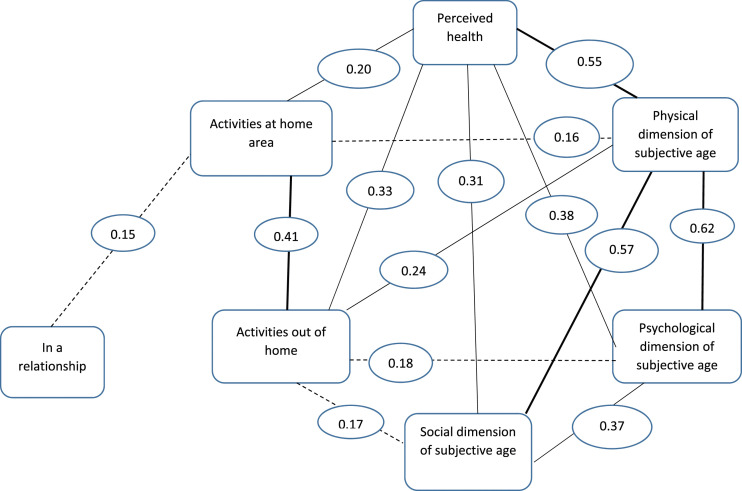
Correlations between variables (all *p*-values < .001). Polychoric correlation is used between the three dimensions of subjective age and perceived health, all others are Spearman’s correlations. Correlation > .30, Correlation > .20 - < .30, Correlation > .10 - < .20.

To illustrate correlations between the variables, bivariate correlation coefficients were calculated, and all associations with *p* < .001 are shown in Figure 1.

## Discussion

This study aimed to explore the associations between perceived health, subjective age, and participation in social activities among older persons (65 years old and older, living in their private homes) in the Bothnia and Åland islands regions in Finland. The present study contributes to the previously identified gap in studies exploring the subjective and social dimensions of healthy aging (Nordmyr, et al., 2020; Tully-Wilson, et al., 2021). There is no single path to “healthy aging.” Therefore, chronological age as well as objective measurements of health status may not be ideal when studying aging and health in gerontological research, as our different pathways and conditions lead to differences in health in later life (Halfon & Forrest, 2018). Overall, previous studies have addressed the importance of looking more closely at the context of everyday life in order to understand the connection between subjective age and health in later life—that is, exploring the experiences and conditions that might influence a person to feel younger or older than their chronological age (Hughes & Touron, 2021).

The results of this study indicate that most of the older respondents seem to feel younger (psychological dimensions of subjective age) than their chronological age, which is similar to what has been reported in several previous studies (e.g., Wettstein, et al., 2023). For instance, in a Norwegian study, almost 87% felt younger than their chronological age (Langballe et al., 2023). This seem to be particularly the case of older women in the present study, as they were more likely than men to report feeling younger than their chronological age. However, most of the older respondents seem to feel physically the same age and also believe that others think they are the same age as their current chronological age (social dimensions of subjective age). The results therefore suggest that the previously described gap between chronological and subjective age may vary depending on which dimension of subjective age is under study or how the survey question about subjective age is formulated.

Subjective age seems to be linked to perceived health in later life. The results of this study show that perceiving oneself as physically, psychologically and socially younger than one’s actual chronological age was associated with better perceived health status and vice versa; an older subjective age predicted worse self-perceived health, which is in line with what has been reported previously regarding subjective age and health (Kotter-Grühn et al., 2015; Spuling et al., 2013; Westerhof et al., 2023). It has been suggested that a younger subjective age predict better health outcomes and can therefore be seen as a health resource (Wettstein et al., 2023). However, a younger perceived age also seems to have negative influences on health over time, as wanting to be younger than one’s chronological age was associated with lower satisfaction and physical functioning (Veenstra et al., 2021). It is also likely that our health status influences perceived age. Various health problems, such as limitations in vision, hearing and incontinence, seem to make older persons feel older than their actual chronological age (Langballe et al., 2023), or at least as less young. Thus, subjective age may be shaped by individual experiences of health. Because, this study focused on subjective dimensions of health, we do not know whether subjective age differed between groups of older persons with and without health problems and whether health conditions and functional limitations influenced the experience of age. Overall, the older persons in this study reported their health as generally good, which may shed light on why few older persons reported feeling older than their actual chronological age. However, previous studies have a highlighted that older people tend to rate their perceived health status as high despite health problems and multimorbidity (Wettstein et al., 2023), highlighting that good perceived health is not equivalent to absence of disease.

The discrepancies between subjective and chronological age and between objective and subjective measures of health have been discussed in previous studies in terms of social comparisons. Sayag and Kavé (2022) explored who older persons tend to compare themselves with when assessing subjective age and self-rated health and found that persons appear to compare themselves primarily with peers, and often with peers who are worse-off health-wise, which may result in reporting a younger subjective age and a better self-rated health. This kind of “downward comparison” may be a way of distancing oneself (consciously or unconsciously) from aging, and in a sense, an existential negotiation with death. Disassociation from chronological age and peers have also been suggested to reflect ageism, as perceiving oneself as young often seems to be associated with something positive, and feeling old as something negative, associated with decline rather than vitality (Gendron et al., 2018).

The study results also show that participating in activities outside the home had statistically significant associations with feeling physically, psychological, and socially younger than the chronological age. Previous studies have found that older persons who feel younger report engaging in activities related to exercise, appearance (Montpare, 2019), and social activities (Takatori et al., 2018), while chronological age has been found to predict, for example, seeking healthcare (Montpare, 2019). Previous studies also highlight that in addition to subjective age also our views of older persons seem to influence our behavior and activities in later life as negative perceptions of older persons and wanting to distance oneself from “the elderly others” may be a reason for non-participation in social and leisure activities for older persons (Saed et al., 2020). Negative views of aging have been suggested to increase the risk of experiencing loneliness and depressive symptoms (Segel-Karpas et al., 2022) and positive perceptions are associated with better health (Tully-Wilson et al., 2021).

Additionally, health status has also been identified as an enabler and barrier to participation in social activities in previous studies (Townsend et al., 2021), and the results of this study further show that the older respondents who participated in activities outside the home showed better perceived health status than the respondents who participated in activities at home or did not participate in any activities. The health status of older individuals may influence participation in social activities and the types of activities in which one can participate (Nivestam et al., 2023), at the same time as activities found meaningful may also promote well-being (WHO, 2021; Nordmyr et al., 2020). Supporting participation would therefore be important to ensure that all older persons, regardless of health status, have equal opportunities to engage in desired activities. However, current social and health services do not seem to support participation in activities outside home sufficiently and tend to overlook the psychosocial needs of older persons (Van Aerschot et al., 2022) and therefore need to be developed. Overall, changing the way we think and feel about older age and later life are important for promoting healthy aging (WHO, 2021) and taking into account subjective perceptions of age and health and recognizing and addressing the heterogeneity of older people is an important step in this process.

### Study strengths and limitations

One of the key remarks to consider when interpretating the results is that the data was collected in the autumn of 2021, when the Covid 19-pandemic was still influencing the organization of activities and events. The virus mitigation measures recommended from the beginning of the pandemic were related to physical distancing from persons outside one’s home and avoiding public spaces and social gatherings. However, even if the recommendations were somewhat more relaxed in 2021 and the Finnish society returned to daily patterns and routines similar to those before the pandemic, older persons in particular may still have taken some precautions when it comes to participating in activities outside their home. This is likely to have influenced the study results as the participation in leisure activities among older persons decreased during the pandemic (Nyqvist et al., 2023). The present study did not explore online activities, which can be seen as an additional limitation. Various types of online services are an important part of many older persons everyday lives and should increasingly be explored alongside “offline” activities to give a more comprehensive picture of daily later life. Additionally, the pandemic might also have changed older persons views of age and health. In retrospect, the pandemic in several ways exposed ageism and related stereotypical images of older people as a homogenous group of frail persons (United Nations, 2021), which seems to have negatively influenced self-perceptions of aging (Kornadt et al., 2021).

Another factor to consider is the survey sample and who the survey may or may not have reached. As the survey was quite an extensive survey (consisting of 92 items), it may have been the case that older persons with health problems did not complete the survey to the same extent as more health resourceful older persons. This should be taken into account when interpreting the results as well as that several of the socio-economic and background variables included showed statistically significant associations to the outcome variables in the analysis—hence, several factors may be influencing the association between subjective age, perceived health, and social activities in later life. The data analysis did not include a variable measuring income level, which is another limitation with the study, as previous studies show a connection between individual economic circumstances and health (e.g., Lai et al., 2021), while other studies suggest that the influences of income on subjective dimensions of health remain unclear (e.g., George, 2010). However, income level is likely to influence participation in activities and should therefore be included in future studies in addition to employment status as older persons working after official retirement age are increasing. Moreover, it is also important to remember that subjective age is not just a personal view—it is influenced by the society and culture in which persons are aging, and therefore, the study context, that is, the regions with both Swedish-speaking and Finnish-speaking populations, should be kept in mind.

Finally, the study is based on cross-sectional data, and one of the main limitations of such a design is that it does not allow statements to be made about the causal relationship between the variables studied. However, the present study also had several strengths that should be considered alongside the limitations raised and discuss in this section. This study is based on a relatively large, high-quality survey encompassing several cohorts of older persons in a certain context. Additionally, unlike many previous studies exploring the link between subjective age and health, this study included several dimensions of subjective age. One-item measurements of subjective age dominate the current research field and a more nuanced approach is needed to advance knowledge. Furthermore, the study also attempts to include social and everyday life activities in the analysis and understanding of subjective age and health in later life, which previously have received less attention.
